# Uncoiling CNLs: Structure/Function Approaches to Understanding CC Domain Function in Plant NLRs

**DOI:** 10.1093/pcp/pcy185

**Published:** 2018-09-06

**Authors:** Adam R Bentham, Rafał Zdrzałek, Juan Carlos De la Concepcion, Mark J Banfield

**Affiliations:** Department of Biological Chemistry, John Innes Centre, Norwich Research Park, Norwich, UK

**Keywords:** Cell death signaling, Coiled-coil domain, NLR, Oligomerization, Plant immunity, Protein–protein interactions

## Abstract

Plant nucleotide-binding leucine-rich repeat receptors (NLRs) are intracellular pathogen receptors whose N-terminal domains are integral to signal transduction after perception of a pathogen-derived effector protein. The two major plant NLR classes are defined by the presence of either a Toll/interleukin-1 receptor (TIR) or a coiled-coil (CC) domain at their N-terminus (TNLs and CNLs). Our knowledge of how CC domains function in plant CNLs lags behind that of how TIR domains function in plant TNLs. CNLs are the most abundant class of NLRs in monocotyledonous plants, and further research is required to understand the molecular mechanisms of how these domains contribute to disease resistance in cereal crops. Previous studies of CC domains have revealed functional diversity, making categorization difficult, which in turn makes experimental design for assaying function challenging. In this review, we summarize the current understanding of CC domain function in plant CNLs, highlighting the differences in modes of action and structure. To aid experimental design in exploring CC domain function, we present a ‘best-practice’ guide to designing constructs through use of sequence and secondary structure comparisons and discuss the relevant assays for investigating CC domain function. Finally, we discuss whether using homology modeling is useful to describe putative CC domain function in CNLs through parallels with the functions of previously characterized helical adaptor proteins.

## Introduction

Plants use intracellular immune receptors to perceive virulence proteins (effectors) secreted to the host cell by microbial pathogens during infection (Dangl and Jones 2001). These receptors generally belong to the superfamily of nucleotide-binding (NB) leucine-rich repeat (LRR) receptors (NLRs) (Kourelis and van der Hoorn 2018), which are also components of mammalian innate immunity pathways (Duxbury et�al. 2016, Jones et�al. 2016, Bentham et�al. 2017, Meunier and Broz 2017). Plant NLR proteins are commonly divided into two classes based on their N-terminal domains: coiled-coil- (CC) containing NLRs (CNLs), and Toll/interleukin-1 receptor- (TIR) containing NLRs (TNLs). Mammalian NLRs typically detect PAMPs (pathogen-associated molecular patterns) (Broz and Monack 2013), although recently NLRP1B was shown to be activated by proteolytic cleavage mediated by bacterial pathogen effectors (Bachovchin et�al. 2018, Sandstrom et�al. 2018). In contrast, plant NLRs have only been shown to respond to specific effector molecules. A recognition event between a plant NLR and pathogen effector typically results in a form of programmed cell death known as the hypersensitive response (HR). This response results in host cell death, but also isolates the pathogen, preventing colonization of the plant and disease (Heidrich et�al. 2012). NLRs offer genetic solutions to preventing disease in crops, and have been widely used in plant breeding programs (Mundt 2018), without requiring the use of costly and unsustainable pesticides. This has stimulated research focused on understanding how these receptors detect effectors and initiate immunity-related signaling.

Plant NLRs have a modular, multidomain architecture, and specific roles for each domain have been described that together contribute to the function of the full receptor (Takken and Goverse 2012).The C-terminal LRR domain has been implicated in direct binding of effectors in some NLRs (Jia et�al. 2000, Dodds et�al. 2006); however, it also appears to have a role in autoinhibition of the receptor preceding effector interaction (Ade et�al. 2007, Faustin et�al. 2007). Activation of immune signaling by NLRs appears to involve ADP/ATP exchange within the central NB domain (also known as NB-ARC in plants) (Tameling et�al. 2006, Williams et�al. 2011, Bernoux et�al. 2016), which may modulate conformational changes within the receptor in response to effector detection (Takken et�al. 2006, Bernoux et�al. 2016). The NB domain of plant NLRs shares similarities with the NACHT domain (the nucleotide-binding domain of mammalian NLRs) which undergoes conformational change in mammalian NLRs, as observed by cryo-electron microscopy (cryo-EM) structures of NLRC4 apoptosomes and the crystal structures of the NB domain of the NLR-like apoptosis protein, APAF1 (Reubold et�al. 2011, Hu et�al. 2015, Zhang et�al. 2015, Tenthorey et�al. 2017). Recently, multiple studies have categorized the function of supplementary domains found only in some NLRs, which may be found attached to the N- or C-terminus of the protein, or even incorporated between the other domains of the receptor (Cesari et�al. 2014, Maqbool et�al. 2015, Wu et�al. 2015, Kroj et�al. 2016, Sarris et�al. 2016, De la Concepcion et�al. 2018). Known as integrated domains (IDs), these domains most probably have their evolutionary origin as host effector targets and are associated with direct effector perception (Cesari et�al. 2014, Baggs et�al. 2017). Finally, located at the N-terminus, is either the TIR domain or the CC domain. Both TIR domains and CC domains are thought to be the receptor modules required for downstream signal transduction post-NLR activation (Takken and Goverse 2012); however, CC domains from a variety of different NLRs have also been implicated in guardee or effector perception (Khan et�al. 2016). The TIR and CC domains divide plant NLRs into the TNL and CNL classes (Meyers et�al. 1999, Meyers et�al. 2003). Research to date has established that the TIR domain has a role in signaling by plant TNLs; however, less is known about signaling by CC domains in CNLs. While TNLs can provide resistance to disease in solanaceous, brassicaceous and other crops, they contribute less to the immune systems of cereals, as their NLR repertoires consist of almost entirely CNLs (Bai et�al. 2002, Meyers et�al. 2002). With cereal crops contributing to approximately 50% of the world’s daily caloric intake (FAO 2003), further research into the signaling capacity of CC domains and CNL function is of high priority.

Although considered the predominant signaling units of NLRs, TIR domains and CC domains are structurally and functionally very different from one another. TIR domains adopt a conserved flavodoxin-like fold consisting of five α-helices surrounding a five-strand β-sheet, as observed in crystal structures of TIR domains from a variety of different plant, animal and bacterial species (Ve et�al. 2015). Signal transduction mediated by plant TIR domains has been intimately linked to their ability to self-associate, with two self-association interfaces formed by the surface-exposed regions of the αA and αE, and αD and αE helices, respectively (Bernoux et�al. 2011, Williams et�al. 2014, Zhang et�al. 2017). In contrast, CC domains are largely helical proteins, and there is some debate concerning their overall structure (discussed in this review). Further, despite a growing number of studies, the function of the CC domain in NLR signaling downstream of effector perception remains unclear.

Here, we review current knowledge of CC domain- and CNL-mediated signaling in plant immunity and highlight some methods for investigating CC domain structure and function. We discuss the current classification of CNLs in the context of function, provide some guidelines on how to design CC domain constructs for structural and functional studies through analyses of sequence and secondary structure, and finally discuss putative functions for the variety of different CC domains found in CNLs.

## Highly Unclassified: Functional Analyses of CC Domains Complicate Current Classifications

Previously, CNLs have been characterized based on motifs in the NB domain, and not the CC domain, as low sequence similarity and the absence of consistent motifs in the CC domain made analyses with resources such as Pfam difficult (Meyers et�al. 1999, Meyers et�al. 2002, Meyers et�al. 2003, Finn et�al. 2016). More recently, there have been an increasing number of studies published focusing on the CC domain, and three major features have emerged that are frequently used to describe their function: (i) their ability to trigger cell death when transiently expressed in model host plants such as *Nicotiana benthamiana*; (ii) the need for self-association to signal cell death; and (iii) the presence/absence of CC-specific motifs, such as the EDVID motif. A result of these studies is that CC domains are frequently grouped into several classes: CC_EDVID_, CC_R_, CC (often referred to as the canonical or classical CC domain; herein referred to as CC_CAN_ for clarity) (Collier et�al. 2011), and the I2-like and SD-CC classes, only found in Solanaceous plants (Hamel et�al. 2016). The CC_EDVID_ class, which includes NLRs such as Sr33, MLA10, Rx, SlNRC4, Rp1-D21 and RGA5, are named for the highly conserved EDVID motif that is suggested to be involved in intramolecular interactions with the NB domain (Rairdan et�al. 2008, Bai et�al. 2012, Wang et al. 2015, Leibman-Markus et al. 2018). The CC_R_ subclass is characterized by NLRs with a CC domain that shares similarity to RPW8 (Collier et�al. 2011); classical/canonical CC_CAN_ domains are the CC domains from NLRs that do not fit into the previous two categories, with examples such as RPS2 and RPS5 (Qi et�al. 2012). CC domains belonging to the SD-CC subclass include a large auxiliary domain N-terminal to the CC domain, known as a solanaceous domain (SD); this class includes the well-characterized NLRs, Sw-5b and Prf (Mucyn et�al. 2006, De Oliveira et�al. 2016). Finally, the I2-like CNL family is centered around CC domains with similarity to the CC domain of the tomato NLR, I2. While also possessing an EDVID motif, I2-like CNLs are different from their CC_EDVID_ counterparts, segregating into their own monophyletic clade (Pan et�al. 2000, Couch et�al. 2006, Rairdan et�al. 2008). CNLs with I2-like CC domains include I2, R3a, L and N' (Collier et�al. 2011, Hamel et�al. 2016).

Recently, it has been found that many NLRs (both TNLs and CNLs) function synergistically either as pairs (Ashikawa et�al. 2008, Narusaka et�al. 2009, Cesari et�al. 2014) or as part of intricate signaling networks (Collier et�al. 2011, Zhu et�al. 2011, Wu et�al. 2017). What role the CC domain of CNLs plays in heterologous pairs and/or NLR networks, for example in mediating oligomerization or signaling, is largely unknown.

While many genes encoding CNLs have been identified in plant genomes, only a handful of studies addressing the function of CNL proteins, and the signaling mechanisms of the CC domain, have been performed. For this review, we selected CNLs for which functional data for their CC domains are available, and included experimental data which have tested at least one of the three following functions: (i) capacity to induce cell death; (ii) ability to self-associate; and (iii) ability to interact with a cofactor (for references, see Fig.�1). This includes three CC domains from the CC_CAN_ and CC_R_ subclasses, four CC domains from the I2-like subclass and the rest comprise CC domains of the CC_EDVID_ subclass. While there have been multiple studies performed on the N-terminal SD-CC domains of Sw-5b and Prf SD-CNLs (Gutierrez et�al. 2010, Saur et�al. 2015, De Oliveira et�al. 2016), there are few data for the CC domain function alone. This creates difficulties when attempting to delineate CC domain functions from the functions of the SD and other N-terminal domains, as in the case of Prf. Therefore, CC domains from SD-CNLs have not be included in the subsequent analyses.

**Fig. 1:**
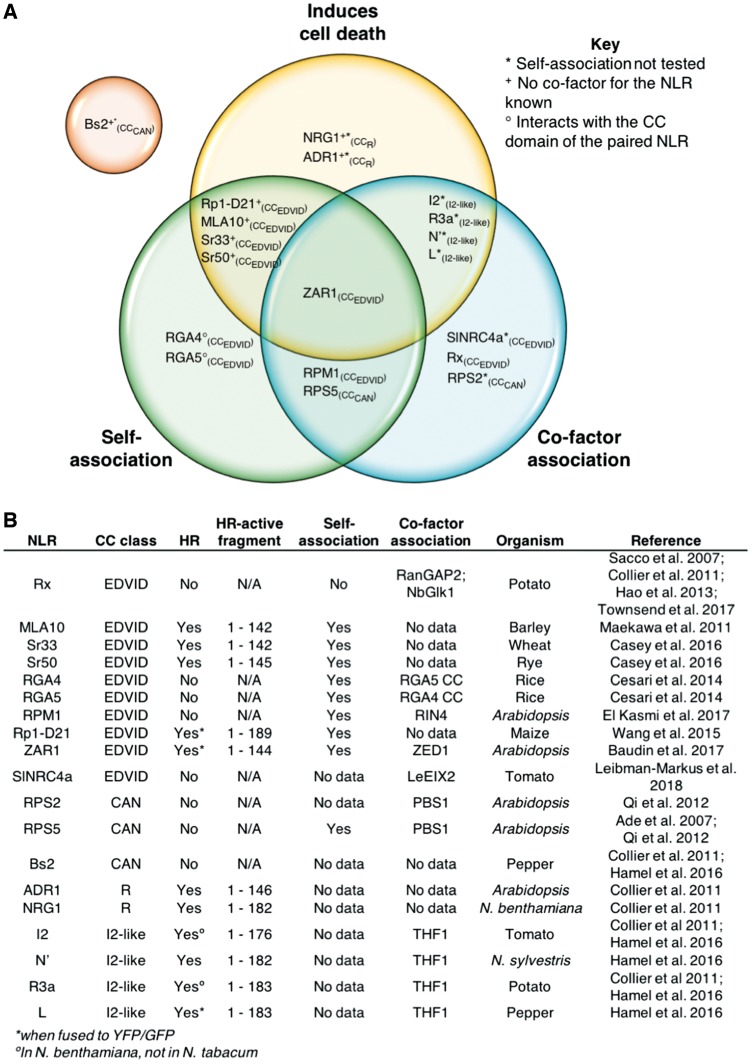
Comparison of known CC domain functions. (A) Venn diagram of the three major CC domain functions assayed: the ability to induce cell death autonomously (yellow), the ability to self-associate (green) and the ability of the CC domain to interact with a cofactor (blue). The selected CC domains analyzed here have been placed in regions that correlate with observed functions. CC domains from all subclasses can be found across all regions of the Venn diagram, demonstrating little correlation between function and subclass. The one exception to this is Bs2 from the CC_CAN_ subclasses, for which there are no observed functions in any of the three categories and is depicted in an orange circle separated from the other CNLs. (B) A table of reported functions of the CC domains analyzed here, accompanied by the studies in which they were observed. As with (A), little correlation can be seen between CC domain function and subclass assignment, with the exception of CC domains that belong to the monophyletic CC_R_ and I2-like subclasses.

When using our defined functional groups, it becomes clear that division into CC_EDVID_, CC_R_ or CC_CAN_ subclasses does not align directly with function (Fig.�1). The exception to this is the CC_R_ (comprising members of the ADR1 family), and I2-like subclasses, which distinctly segregate in sequence and function from other CNLs (Collier et�al. 2011, Hamel et�al. 2016).

The diverse functional groupings of CC domains within the single CC_EDVID_ subclass are the clearest (Fig.�1). CC_EDVID_ domains differ in their ability to self-associate, signal cell death and directly interact with a cofactor. Using the RPM1, MLA10 and Rx CC domains as examples, several subclass contradictions can be observed based on reported functions (Rairdan et�al. 2008, Casey et�al. 2016, El Kasmi et�al. 2017). The CC domains of MLA10, RPM1 and Rx are all of the CC_EDVID_ type, but only the MLA10 CC domain is capable of autonomously signaling cell death (Rairdan et�al. 2008, Casey et�al. 2016, El Kasmi et�al. 2017). Much like MLA10, the RPM1 CC domain self-associates; however, RPM1 CC does not autonomously signal cell death (El Kasmi et�al. 2017), whereas the Rx CC domain does not signal cell death or self-associate (in the 1–122 CC domain construct only) (Moffett et�al. 2002, Casey et�al. 2016). Finally, the RPM1 and Rx CC domains, but not the MLA10 CC domain, interact with a cofactor that is essential for function for the receptor (Moffett et�al. 2002, Sacco et�al. 2007, Bai et�al. 2012, El Kasmi et�al. 2017). It is worth noting that the 1–144 construct of the Rx CC domain has been observed to form large homomeric protein complexes in size-exclusion chromatography (SEC; Townsend et�al. 2018); however, with a lack of biophysical analyses it is difficult to determine whether these complexes are representative of an ordered oligomeric assembly or simply aggregation. Regardless, the different functions described above show that caution must be applied when addressing CC domain function in the context of the CC_EDVID_ classification. While the EDVID motif has been shown to have a role in mediating interactions between the CC domain and the NB domain (Rairdan et�al. 2008, Bai et�al. 2012), its presence alone should not be taken as an indicator of CC domain (or NLR) function, as described here.

A further example highlights shared CC domain functions that span the previously described classes.

RPM1 and RPS5 CC domains share similar functions, with both capable of self-association and cofactor interactions (RPM1 with RIN4, and RPS5 with PBS1), despite belonging to the CC_EDVID_ and CC_CAN_ subclasses, respectively (Ade et�al. 2007, El Kasmi et�al. 2017). Moreover, neither RPM1 nor RPS5 CC domains are capable of independently signaling cell death. There are several other examples of CC domains from different classes that do not signal cell death but interact with a guardee/cofactor, including Rx and RPS2 (Rairdan et�al. 2008, Qi et�al. 2012).

Taken together, these observations highlight that the commonly used classifications of CC domains are not especially useful for confidently defining function. Therefore, care should be taken if these classifications are used when designing functional studies for CC domains from uncharacterized NLRs. The differences in CC domain function, coupled with the small sample size of proteins studied to date, serves to highlight the difficulties in forming classes, and consequently, prediction of putative functions for these proteins based on sequence. One limiting factor in characterizing the function of CC domains in plant NLRs is the difficulty in assigning domain boundaries, and therefore the appropriate design of constructs to analyze. Next, we give a short guide on how to analyze the protein sequences of CC domains with the goal of designing experiments to assay their function robustly.

## Predicting CC Domain Boundaries with Sequence and Secondary Structure Prediction to Guide Functional Studies

Due to a lack of knowledge, it is frequently necessary to make subjective decisions about the boundaries of protein domains when attempting to assess function. This can result in inappropriate constructs that complicate functional annotation. One potential point for error is to use domain boundaries based on homology or similarity to previously assayed proteins. For example, in TIR domains from plant NLRs, despite the core flavodoxin fold being conserved in the protein (Ve et�al. 2015), there is variation in the extent of the domains, and surrounding regions, that are required for in planta cell death phenotypes (Bernoux et�al. 2011, Williams et�al. 2014, Schreiber et�al. 2016). In this case, applying knowledge of construct boundaries from one protein to study the function of another would result in inappropriate conclusions. The same considerations should be applied when designing constructs to assay the function of CC domains, to avoid potential misrepresentation of protein function.

As, to date, the explicit function of CC domains in plant NLRs remains undefined, any initial constructs designed to assay activity should be overly inclusive, preferably spanning the region of the NLR from the N-terminus to the beginning of the NB domain (the boundaries of which can be better defined). These constructs should then be assayed for cell death signaling in planta, cofactor association and self-association to establish a functional reference point before, perhaps, trying to delimit minimal regions required for function (however, it is worth noting that additional residues at the C-terminus of signaling domains has been observed to cause inhibition of signaling in planta (Bernoux et�al. 2011)). Not all CC domains studied to date autonomously signal cell death in planta, and therefore a non-cell death-inducing construct does not imply a lack of biological relevance. Care must be exercised when interpreting assays with new CC domain constructs, and best practice would include trialing several CC domain constructs before making conclusions concerning function.

Two of the more useful, and extensively used, tools for assessing construct boundaries are secondary structure prediction and Pfam domain analysis (Finn et�al. 2016). In many cases, Pfam is an excellent resource for initial identification of regions of interest; however, it can be unsuitable for guiding bespoke domain boundaries. It is well established that Pfam struggles with CC domain identification (Meyers et�al. 2003), therefore it is advisable to include the amino acid sequence of CC domains from the first N-terminal residue of the NLR up to the first Pfam predicted residue of the NB domain when designing initial CC domain constructs. As an example, Pfam analysis of the NLR Sr33 defines the CC domain as encompassing residues 6–134; however, structure/function studies have revealed that residues 1–142 are required for the cell death phenotype, and any less prevents this activity (Casey et�al. 2016, Cesari et�al. 2016). Hence, were the Sr33 CC domain boundaries defined solely by Pfam, and subsequently used as the basis for construct design, this would generate a protein that does not signal cell death, potentially leading to the loss of biologically relevant information. Additional sequence analysis tools should be used when designing CC domain constructs shorter than the N-terminus of the NLR to the start of the NB domain. As shown in Fig.�2, the secondary structure composition of CC domains greatly varies, even within subclasses, and therefore care must be taken when designing constructs.

**Fig. 2:**
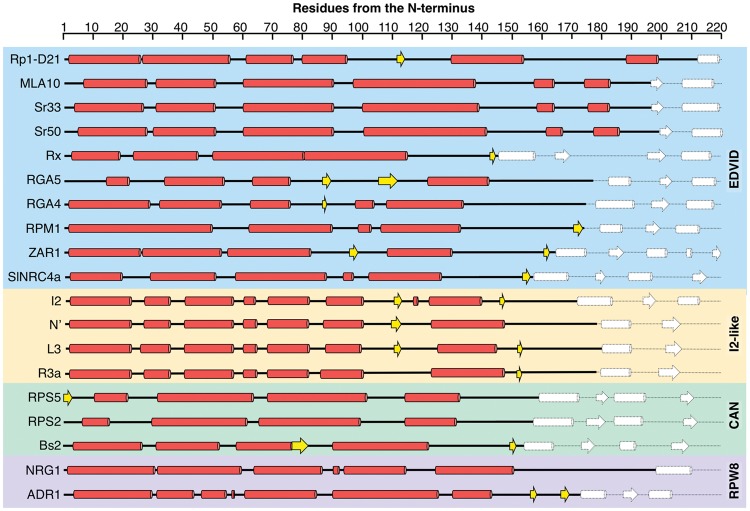
Secondary structure alignment of CC domains. The first 220 amino acids of all selected NLRs were subjected to the PSIPRED (Buchan et al. 2013) secondary structure prediction server and sorted by CC domain subclass. The CC domains assigned to the CC_EDVID_ subclass are in the blue box, with the CC domains of the I2-like, CC_CAN_ and CC_R_ in yellow, green and purple boxes, respectively. The predicted α-helices and β-strands of the CC domains are shown as red cylinders and yellow arrows, respectively. The secondary structures of the NB-ARC domain are represented by white shapes with dashed lines. The start of the NB-ARC domains was predicted with Pfam (Finn et al. 2016). Despite all being largely helical, it is clear that the position of the secondary structures in CC domains varies greatly between NLRs.

Programs such as COILS and PSIPRED, as well as 3-D homology modeling servers such as Phyre2 and I-Tasser, run a secondary structure prediction as a part of their pipelines (Lupas et�al. 1991, Buchan et�al. 2013, Kelley et�al. 2015, Yang and Zhang 2015). In general, these programs predict the position of α-helices and β-strands, accompanied by a confidence score. This knowledge is very valuable as keeping protein secondary structure units intact, without partial removal or truncation, will probably be important for protein stability. In the context of structural biology, secondary structure prediction is also useful to avoid long, disordered regions in the protein, which can result in solubility issues, promote aggregation or make crystallization difficult (Dong et al. 2007). While the prediction of secondary structure is a very useful tool, much like Pfam, domain boundaries should not solely rely on the outputs of this software, but rather be used as a guide. Best practice would be to generate several CC constructs, with additional residues at the C-terminus of the last secondary structure to ensure that any predicted α-helix or β-strand is fully covered.

## Assaying CC Domain Function: Comparing Results and Understanding Technique Limitations

Generalizing plant NLR CC domain function has proven challenging. As discussed previously, the ability to cause cell death upon heterologous expression in model plants, self-association and whether the domain interacts with a cofactor are the most commonly assigned activities. Each of these activities are informative concerning CC domain function, but the conclusions can be subjective, in particular where a lack of activity is observed, as this could just be due to suboptimal experimental design.

After assembling suitable constructs through incorporating best estimates of domain boundaries, assays to assess the CC domain functions described above can be employed. The in planta ‘HR assay’, often performed in heterologous hosts such as *N. benthamiana* or *N. tabacum*, is used as an indicator of cell death signaling consistent with plant immunity pathways. As it is less likely that negative (lack of cell death) responses will be reported in the literature, it is difficult to assess how common it is for CC domains to lead to cell death on expression. Good examples of how to use the HR assay to evaluate CC domain autoactivity are found in Bai et�al. (2012), Cesari et�al. (2016), Casey et�al. (2016) and El Kasmi et�al. (2017). In each of these studies, several CC domain constructs were tested with different domain boundaries to explore the extent required for signaling, or whether signaling was not observed, as in the case of RPM1 (El Kasmi et�al. 2017).

To study CC domain self-association, or association with cofactors, yeast two-hybrid (Y2H), co-immunoprecipitation (CoIP) and analytical SEC (also known as gel filtration) have all been extensively used. While powerful techniques, Y2H and CoIP (from plant tissue, usually after co-expression in *N. benthamiana*) suffer from false negatives and positives due to the context of the assay. Y2H assays can positively report the interaction of two proteins, but this may not be biologically relevant, and false negatives can occur from inhibition of interactions by the reporter fusions, or because a partner of the interaction (e.g. a ‘bridging molecule’) is missing in yeast. While CoIP assesses associations derived from plant tissue (that can also be affected by ‘bridging molecules’), extraction conditions may both positively promote and negatively influence interactions between the proteins of interest. In cases where differences are observed between interactions in Y2H and CoIP analyses, further experiments should be conducted.

One such technique is SEC, but this is a purely in vitro assay that relies on heterologous expression and purification of the protein(s) of interest, mostly commonly *Escherichia coli*. SEC reports protein shape, which is correlated with size, and can be used to study protein self-association or complex formation by comparison of retention times with known standards. CC domain self-association in vitro has been observed as weak and transient, and SEC alone may not have the resolution required to measure self-association confidently (Casey et�al. 2016). The nature of oligomeric, self-associating complexes (or complexes comprised of different proteins) observed in SEC can be further investigated using other in-solution biophysical techniques, such as small-angle X-ray scattering (SAXS), or multiangle laser light scattering (MALS). These techniques derive accurate measurements of the average molecular mass of the sample, but do require specialist equipment that may not be routinely available. Examples of MALS and SAXS analyses with the plant immunity field include those of CC domains (Casey et�al. 2016), characterization of TIR domain self-association (Bernoux et�al. 2011, Williams et�al. 2014, Zhang et�al. 2017) and investigation of heterocomplex formation between the RxLR effector PexRD54 and the host autophagy-related protein, ATG8 (Maqbool et�al. 2016). Furthermore, the stoichiometry of protein complexes can be difficult to determine by SEC, and additional techniques, such as MALS, surface plasmon resonance (SPR) or isothermal titration calorimetry (ITC), can be used to assess stoichiometry of the complex and (for the latter two) determine binding affinities when investigating heterocomplexes (Maqbool et�al. 2015, De la Concepcion et�al. 2018).

Overcoming the limitations of individual techniques to study plant NLR CC domain function requires multiple approaches. One of the best predictors of protein function is 3-D structure, and currently structural data on CC domains are limited. For example, to date, there is no structure of a plant NLR CC domain that fully covers a construct that induces cell death in model plants. In the next section, we assess the current understanding of CC domain structure, and ask whether homology modeling is useful to provide insight into plant NLR CC domain function, specifically to inform further experiments.

## CC Domain Structure: A Protein Interaction Scaffold or Another Function?

The Sr33 CC domain [determined by nuclear magnetic resonance (NMR)], and the MLA10 and Rx CC domains (determined by X-ray crystallography), are the only structures of plant CC domains available to date (Maekawa et�al. 2011, Hao et�al. 2013, Casey et�al. 2016). The Sr33 and Rx CC domains comprise a four-helix bundle fold (Hao et�al. 2013, Casey et�al. 2016). In contrast, in the crystal structure of the MLA10 CC domain, the protein forms an obligate dimer made up of two helix–loop–helix monomers (Maekawa et�al. 2011) (Fig.�3A). Interestingly, while the structure of the MLA10 CC domain does not resemble the structures of Rx CC, or of MLA10’s ortholog Sr33, a comparative study of the biophysical characteristics of the three CC domains reveals that they probably share the same four-helix bundle fold in solution (Casey et�al. 2016). This raises intriguing questions on the helix–loop–helix dimer structure of MLA10. It is possible that this conformation represents either a crystallographic artifact, or a biologically significant oligomerization state that may also be observed for other plant NLR CC domains (El Kasmi and Nishimura 2016).

**Fig. 3:**
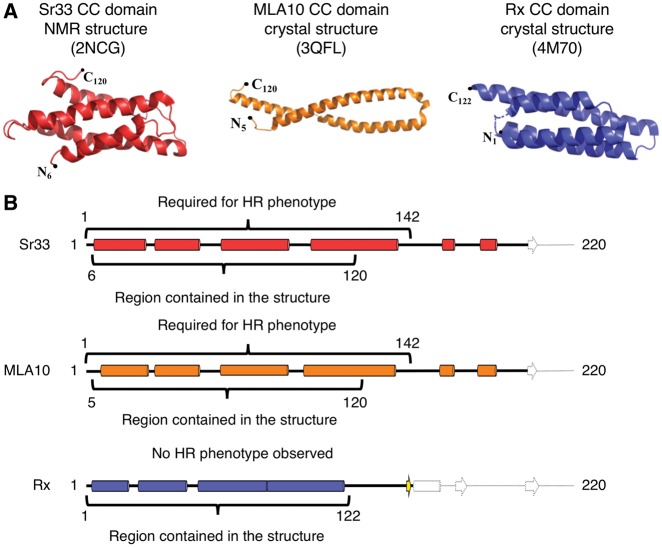
Currently known experimentally determined CC domain structures and their predicted secondary structure. (A) The three-dimensional structures of Sr33, MLA10 and Rx CC domains. The Sr33 and Rx CC domains maintain a monomeric four-helix bundle fold. However, MLA10 CC forms an extended helix–loop–helix structure and is thought to form an obligate dimer. Despite the differences in structures, Sr33 and MLA10 are orthologs (Periyannan et al. 2013). (B) Secondary structure predictions of the Sr33, MLA10 and Rx CC domains. Highlighted on the secondary structures are the regions represented in the crystal structures compared with the regions required for inducing an HR-like phenotype in model plants. The structures of the MLA10 and Sr33 CC domains do not represent functional HR signaling units, which is likely to be compromised by the truncation of the fourth α-helix, as seen in the secondary structure prediction. The Rx CC domain structure comprises an entire four-helix bundle; however, this region does not display autonomous cell death signaling in model plants.

As previously mentioned, none of the existing plant NLR CC domain structures represents functional signaling units, at least in the context of induced cell death in model plants (Fig.�3). For both the Sr33 and MLA10 CC domains, an additional 22 residues are required at the C-terminus for this activity that were not included in the expressed protein for structural studies (Casey et�al. 2016, Cesari et�al. 2016). Secondary structure predictions of Sr33 and MLA10 suggest that these additional 22 residues are involved in the completion of a fourth α-helix (Fig.�3B), and it was shown that these additional residues are also necessary for the self-association of the MLA10 and Sr33 CC domains (Casey et�al. 2016, Cesari et�al. 2016). It is noteworthy that the structure of the Rx CC domain includes the entirety of a predicted four-helix bundle, but this construct does not induce cell death. Therefore, it would appear that the presence of the entire fourth α-helix in the Rx CC is not sufficient for cell death activity.

In the absence of easy access to structural information, homology modeling offers an opportunity to gain insight into protein structure/function relationships, but should always be used with caution to prevent falling into the ‘functional homology trap’ (Mor�ra et�al. 1994, Lahm et�al. 2003, Launay and Simonson 2008). Specific to homology modeling plant NLR CC domains, with only three template structures available there is a limited pool of information that can lead to bias in the outputs. For this reason, we have removed homology models from the analysis below when these structures have been used as templates.

We took each of the CC domains detailed in Fig.�1, and submitted the sequences to the protein structure prediction server PHYRE2 (Kelley et�al. 2015). Intriguingly, two structures were consistently identified as reasonable templates for homology modeling of these CC domains (Fig.�4A, B). For NRG1 and ADR1, the highest confidence hit was to the NMR structure of the N-terminal domain of the mixed-lineage kinase domain-like (MLKL) protein, and for all other CC domains the highest confidence hit was the CARD (caspase-activation and recruitment domain) of the *Caenorhabditis elegans* CED-4. The similarity between the CC domains of MLKL/CED-4 CARD and those of plant NLRs, as predicted by Phyre2, may be useful to inform further studies of plant NLR CC domain function.

**Fig. 4:**
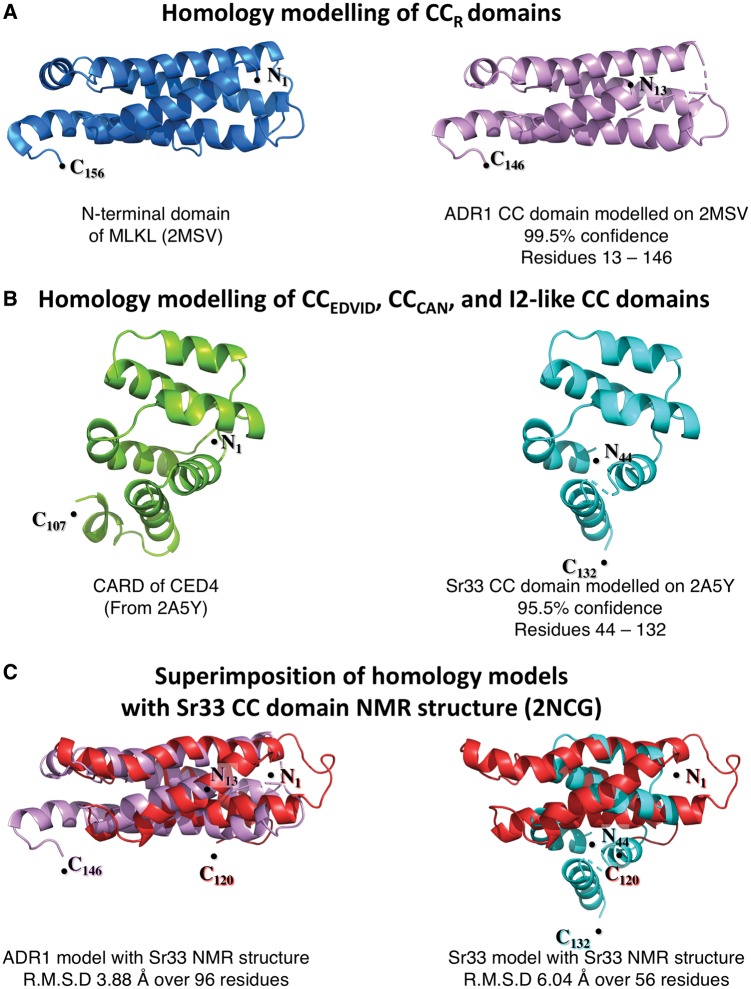
Homology modeling of CC domains compared with experimentally determined structural data. Initial homology models were generated for each of the CC domains previously analyzed in Figs. 1 and 2. For each of the CC domains, sequences from the distal N-terminus to the start of the NB-ARC domain (as predicted by Pfam) were used to generate the models. Only two templates were consistently selected for modeling by PHYRE2 (when excluding the MLA10 CC domain crystal structure, PDB: 3QFL). These were the NMR structure of the N-terminal domain of MLKL (PDB: 2MSV) for CC domains of the CC_R_ subclass, and the crystal structure CARD domain of CED-4 (PDB: 2A5Y) for all other CC domains from the CC_EDVID_, CC_CAN_, and I2-like subclasses. Models of the ADR1 and Sr33 CC domains were generated by one to one threading as representatives of the CC domain homology models based on the two templates, 2MSV and 2A5Y, using domain boundaries defined by secondary structure and cell death signaling capacity in planta. (A) The homology model of the ADR1 CC domain (right, in violet) is shown as a representative of the CC_R_ subclass. Although only sharing 17% sequence identity to MLKL (structure on the left, shown in blue), the model generated covered 89% of the query sequence, modeling residues 13–146 (133 of 150 residues input) with 99.5% confidence. (B) The homology model of the Sr33 CC domain (right, shown in cyan), chosen as the representative of the CC_EDVID_, CC_CAN_ and I2-like subclasses. The Sr33 CC domain shares 10% sequence identity with the CED-4 CARD (structure on the left, shown in green), and the homology model generated covers 61% of the query sequence modeling residues 44–132 (88 of 144 residues input) with a confidence of 95.5%. (C) Left: superimposition of the CC domain homology model of ADR1 (violet) with the NMR structure of the Sr33 CC domain (red) using combinatorial extension. Of the 133 residues modeled, 96 residues of the ADR1 homology model could be superimposed on the Sr33 CC domain NMR structure with a root mean square deviation of 3.88 �. This shows similarity in the overall fold, and suggests that the ADR1 CC homology model may represent a reasonable structure for this CC domain. Right: superimposition of the Sr33 CC domain homology model (cyan) with the NMR structure of the Sr33 CC domain (red) using combinatorial extension. The Sr33 homology model does not represent an accurate depiction of the Sr33 CC domain as seen by the poor superimposition on the Sr33 NMR structure with 56 of the 88 modeled residues superimposed with a root mean square deviation of 6.04 �. This is despite the high confidence score assigned by PHYRE2 to the model.

MLKL is required in the activation of necroptosis, an auxiliary form of cell death thought to be triggered after suppression of apoptosis. The four-helix bundle region of MLKL was necessary for the insertion of the protein into the plasma membrane post-oligomerization, causing pore formation and resulting in the collapse of cell integrity (Su et�al. 2014). Further, the four-helix bundle of MLKL shares similar biochemical properties with CC domains, being highly amphipathic with a highly charge solvent-exposed surface and hydrophobic core (Su et�al. 2014, Casey et�al. 2016, Cesari et�al. 2016). The homology model of the ADR1 CC domain generated from MLKL with PHYRE2 has a high confidence score of 99.5% over the 133 residues able to be modeled (13–146; input 1–150) (Fig.�4A). Intriguingly, the MLKL structure (PDB: 2MSV) was the strongest hit, higher than that of MLA10, which is found ubiquitously as the ‘best template’ when modeling CC domains. Combinatorial extension- (CE) based superimposition of the ADR1 homology model with the four-helix bundle Sr33 NMR structure demonstrates a clear structural similarity, with root mean square deviation of 3.88 � over 96 residues (Fig.�4C). This, combined with the similar biochemical properties between MLKL and CC domains, is indicative of this homology model possibly representing a reasonable approximation of the CC domain structure. However, similarity to the N-terminus of MLKL is only observed for the CC_R_ domains.

As previously mentioned, other homology models of CC domains (all of the CC_EDVID_, CC_CAN_ and I2-like subclasses) were generated using the CARD of CED-4 (PDB: 2A5Y) as a template by the prediction program. The structural folds of CARD domains, including that of CED-4 (Fig.�4B), are known as death domains (DDs), and these are most often observed as the signaling domains of proteins involved in animal immunity (including NLRs) and apoptotic pathways (Vajjhala et�al. 2017). Although often highly divergent amino acid sequences, DDs share a globular structure consisting of six antiparallel amphipathic helices that form a helical bundle. DDs form homotypic interactions with other DD-containing proteins, and regularly assemble into larger oligomeric structures through induced proximity promoted by the oligomerization of the C-terminal domains as seen in APAF-1 and CED-4 apoptosomes (Qi et�al. 2010, Zhou et�al. 2015). The theoretical structural homology of the CC_EDVID_ and CC_CAN_ classes to DDs fits well with current models of plant NLR activation, in which oligomerization may be required for signal transduction by the N-terminal domains (Duxbury et�al. 2016, Bentham et�al. 2017).

While appealing observations, the homology models based on the CARD domain of CED-4 are problematic, and highlight the potential pitfalls of structural modeling. Using the Sr33 CC domain homology model of as an example, CE-based superimposition of the Sr33 CC homology model on the Sr33 CC NMR structure reveals that the homology model does not conform to the known structure (Fig.�4 C). It is important to note that the confidence of the Sr33 CC domain homology model was high (95%), much like the homology model of ADR1; however, of the 88 residues that were modeled (44–132; input 1–144), only 56 could be aligned to the Sr33 NMR structure with a root mean square deviation of 6.04 � (Fig.�4C). Therefore, care must be taken if using confidence scores alone to assess model quality. For structural modeling of CC domains to be sufficient to guide functional studies, additional experimentally derived structures are required.

## Concluding Remarks

In this review we have summarized the current understanding of CC domain structure/function in plant CNLs, and discussed the limitations of CC domain classifications. We highlight the importance of careful consideration in defining CC domain boundaries prior to structural and functional analysis to avoid unintended loss of activity (e.g. cell death induction, ability to self-associate) and maximize biological relevance. From the work presented here, it is clear that significant gaps remain in our knowledge of how CC domains of plant CNLs are involved in transducing defense-related signaling in cells in response to pathogens. Further studies are required to understand how these domains contribute to disease resistance in some of the world’s critical food crops.

## Funding

This work was supported by the Biotechnology and Biological Sciences Research Council [BBSRC; grant Nos. BB/P012574, BB/M02198X]; the European Research Council [ERC; proposals 743165, 669926]; and the John Innes Foundation.

## Disclosures

The authors have no conflicts of interest to declare.

## Abbreviations

Y2H: , Yeast two-hybrid; CC, coiled-coil; CNL, CC domain-containing NLR; DD, death domain; CoIP, co-immunoprecipitation; HR, hypersensitive response; ID, integrated domain; ITC, isothermal titration calorimetry; LRR, leucine-rich repeat; MALS, multiangle laser light scattering; NACHT, NAIP, CIITA, HET-E and TEP1; NB, nucleotide-binding (domain); NB-ARC, nucleotide-binding domain shared by APAF-1, certain R gene products and CED-4; NLR, nucleotide-binding leucine-rich repeat receptor; NMR, nuclear magnetic resonance; PAMP, pathogen-associated molecular pattern; SAXS, small-angle X-ray scattering SEC size-exclusion chromatography; SPR, surface plasmon resonance; TIR, Toll/interleukin-1 receptor; TNL, TIR domain-containing; NLR

## References

[pcy185-B1] Ade J., DeYoung B.J., Golstein C., Innes R.W. (2007) Indirect activation of a plant nucleotide binding site-leucine-rich repeat protein by a bacterial protease. Proc. Natl. Acad. Sci. USA 104: 2531–2536. 17277084 10.1073/pnas.0608779104 PMC1790868

[pcy185-B2] Ashikawa I., Hayashi N., Yamane H., Kanamori H., Wu J., Matsumoto T. (2008) Two adjacent nucleotide-binding site-leucine-rich repeat class genes are required to confer Pikm-specific rice blast resistance. Genetics 180: 2267–2276. 18940787 10.1534/genetics.108.095034 PMC2600957

[pcy185-B3] Bachovchin D., Chui A.J., Okondo M., Rao S., Gai K., Griswold A., et al (2018) N-terminal degradation activates the Nlrp1b inflammasome. bioRxiv doi.org/10.1101/317826

[pcy185-B4] Baggs E., Dagdas G., Krasileva K.V. (2017) NLR diversity, helpers and integrated domains: making sense of the NLR IDentity. Curr. Opin. Plant Biol. 38: 59–67. 28494248 10.1016/j.pbi.2017.04.012

[pcy185-B5] Bai S., Liu J., Chang C., Zhang L., Maekawa T., Wang Q., et al (2012) Structure–function analysis of barley NLR immune receptor MLA10 reveals its cell compartment specific activity in cell death and disease resistance. PLoS Pathog. 8: e1002752. 22685408 10.1371/journal.ppat.1002752 PMC3369952

[pcy185-B6] Bai J., Pennill L.A., Ning J., Lee S.W., Ramalingam J., Webb C.A., et al (2002) Diversity in nucleotide binding site-leucine-rich repeat genes in cereals. Genome Res. 12: 1871–1884. 12466291 10.1101/gr.454902 PMC187567

[pcy185-B7] Bentham A., Burdett H., Anderson P.A., Williams S.J., Kobe B. (2017) Animal NLRs provide structural insights into plant NLR function. Ann. Bot. 119: 827–702. 27562749 10.1093/aob/mcw171 PMC5378188

[pcy185-B8] Bernoux M., Burdett H., Williams S.J., Zhang X., Chen C., Newell K., et al (2016) Comparative analysis of the flax immune receptors L6 and L7 suggests an equilibrium-based switch activation model. Plant Cell 28: 146–159. 26744216 10.1105/tpc.15.00303 PMC4746675

[pcy185-B9] Bernoux M., Ve T., Williams S., Warren C., Hatters D., Valkov E., et al (2011) Structural and functional analysis of a plant resistance protein TIR domain reveals interfaces for self-association, signaling, and autoregulation. Cell Host Microbe 9: 200–211. 21402359 10.1016/j.chom.2011.02.009 PMC3142617

[pcy185-B10] Broz P., Monack D.M. (2013) Newly described pattern recognition receptors team up against intracellular pathogens. Nat. Rev. Immunol. 13: 551. 23846113 10.1038/nri3479

[pcy185-B11] Buchan D.W.A., Minneci F., Nugent T.C.O., Bryson K., Jones D.T. (2013) Scalable web services for the PSIPRED protein analysis workbench. Nucleic Acids Res. 41: W349–W357. 23748958 10.1093/nar/gkt381 PMC3692098

[pcy185-B12] Casey L.W., Lavrencic P., Bentham A.R., Cesari S., Ericsson D.J., Croll T., et al (2016) The CC domain structure from the wheat stem rust resistance protein Sr33 challenges paradigms for dimerization in plant NLR proteins. Proc. Natl. Acad. Sci. USA 113: 12856–12861. 27791121 10.1073/pnas.1609922113 PMC5111715

[pcy185-B13] Cesari S., Bernoux M., Moncuquet P., Kroj T., Dodds P.N. (2014) A novel conserved mechanism for plant NLR protein pairs: the ‘integrated decoy’ hypothesis. Front. Plant Sci. 5: 606. 25506347 10.3389/fpls.2014.00606 PMC4246468

[pcy185-B14] Cesari S., Moore J., Chen C., Webb D., Periyannan S., Mago R., et al (2016) Cytosolic activation of cell death and stem rust resistance by cereal MLA-family CC–NLR proteins. Proc. Natl. Acad. Sci. USA 113: 10204–10209. 27555587 10.1073/pnas.1605483113 PMC5018743

[pcy185-B15] Collier S.M., Hamel L.P., Moffett P. (2011) Cell death mediated by the N-terminal domains of a unique and highly conserved class of NB-LRR protein. Mol. Plant Microbe Interact. 24: 918–931. 21501087 10.1094/MPMI-03-11-0050

[pcy185-B16] Couch B.C., Spangler R., Ramos C., May G. (2006) Pervasive purifying selection characterizes the evolution of I2 homologs. Mol. Plant Microbe Interact. 19: 288–303. 16570659 10.1094/MPMI-19-0288

[pcy185-B17] Dangl J.L., Jones J.D.G. (2001) Plant pathogens and integrated defence responses to infection. Nature 411: 826–833. 11459065 10.1038/35081161

[pcy185-B18] De la Concepcion J.C., Franceschetti M., Maqbool A., Saitoh H., Terauchi R., Kamoun S., et al (2018) Polymorphic residues in rice NLRs expand binding and response to effectors of the blast pathogen. Nat. Plants 4: 576–585. 29988155 10.1038/s41477-018-0194-x

[pcy185-B19] De Oliveira A.S., Koolhaas I., Boiteux L.S., Caldararu O.F., Petrescu A.J., Oliveira Resende R., et al (2016) Cell death triggering and effector recognition by Sw-5 SD-CNL proteins from resistant and susceptible tomato isolines to Tomato spotted wilt virus. Mol. Plant Pathol. 17: 1442–1454. 27271212 10.1111/mpp.12439 PMC6638320

[pcy185-B20] Dodds P.N., Lawrence G.J., Catanzariti A.M., Teh T., Wang C.I., Ayliffe M.A., et al (2006) Direct protein interaction underlies gene-for-gene specificity and coevolution of the flax resistance genes and flax rust avirulence genes. Proc. Natl. Acad. Sci. USA 103: 8888–8893. 16731621 10.1073/pnas.0602577103 PMC1482673

[pcy185-B21] Dong A., Xu X., Edwards A.M. Midwest Center for Structural Genomics; Structural Genomics Consortium Chang C., et al (2007) In situ proteolysis for protein crystallization and structure determination. Nat. Methods 4: 1019. 17982461 10.1038/nmeth1118 PMC3366506

[pcy185-B22] Duxbury Z., Ma Y., Furzer O.J., Huh S.U., Cevik V., Jones J.D.G., et al (2016) Pathogen perception by NLRs in plants and animals: parallel worlds. Bioessays 38: 769–781. 27339076 10.1002/bies.201600046

[pcy185-B23] El Kasmi F., Chung E.-H., Anderson R.G., Li J., Wan L., Eitas T.K., et al (2017) Signaling from the plasma-membrane localized plant immune receptor RPM1 requires self-association of the full-length protein. Proc. Natl. Acad. Sci. USA 114: E7385–E7394. 28808003 10.1073/pnas.1708288114 PMC5584451

[pcy185-B24] El Kasmi F., Nishimura M.T. (2016) Structural insights into plant NLR immune receptor function. Proc. Natl. Acad. Sci. USA 113: 12619–12621. 27803318 10.1073/pnas.1615933113 PMC5111699

[pcy185-B25] FAO (2003) World Agriculture: Towards 2015/2030. An FAO Perspective. Food and Agriculture Organization of the United Nations. http://www.fao.org/docrep/005/y4252e/y4252e04b.htm.

[pcy185-B26] Faustin B., Lartigue L., Bruey J.M., Luciano F., Sergienko E., Bailly-Maitre B., et al (2007) Reconstituted NALP1 inflammasome reveals two-step mechanism of caspase-1 activation. Mol. Cell 25: 713–724. 17349957 10.1016/j.molcel.2007.01.032

[pcy185-B27] Finn R.D., Coggill P., Eberhardt R.Y., Eddy S.R., Mistry J., Mitchell A.L., et al (2016) The Pfam protein families database: towards a more sustainable future. Nucleic Acids Res. 44: D279–D285. 26673716 10.1093/nar/gkv1344 PMC4702930

[pcy185-B28] Gutierrez J.R., Balmuth A.L., Ntoukakis V., Mucyn T.S., Gimenez-Ibanez S., Jones A.M.E., et al (2010) Prf immune complexes of tomato are oligomeric and contain multiple Pto-like kinases that diversify effector recognition. Plant J. 61: 507–518. 19919571 10.1111/j.1365-313X.2009.04078.x

[pcy185-B29] Hamel L.-P., Sekine K.-T., Wallon T., Sugiwaka Y., Kobayashi K., Moffett P. (2016) The chloroplastic protein THF1 interacts with the coiled-coil domain of the disease resistance protein N′ and regulates light-dependent cell death. Plant Physiol. 171: 658–674. 26951433 10.1104/pp.16.00234 PMC4854715

[pcy185-B30] Hao W., Collier S.M., Moffett P., Chai J. (2013) Structural basis for the interaction between the potato virus X resistance protein (Rx) and its cofactor ran GTPase-activating protein 2 (RanGAP2). J. Biol. Chem. 288: 35868–35876. 24194517 10.1074/jbc.M113.517417 PMC3861636

[pcy185-B31] Heidrich K., Blanvillain-Baufum� S., Parker J.E. (2012) Molecular and spatial constraints on NB-LRR receptor signaling. Curr. Opin. Plant Biol. 15: 385–391. 22503757 10.1016/j.pbi.2012.03.015

[pcy185-B32] Hu Z., Zhou Q., Zhang C., Fan S., Cheng W., Zhao Y., et al (2015) Structural and biochemical basis for induced self-propagation of NLRC4. Science 350: 399–404. 26449475 10.1126/science.aac5489

[pcy185-B33] Jia Y., McAdams S.A., Bryan G.T., Hershey H.P., Valent B. (2000) Direct interaction of resistance gene and avirulence gene products confers rice blast resistance. EMBO J. 19: 4004–4014. 10921881 10.1093/emboj/19.15.4004 PMC306585

[pcy185-B34] Jones J.D.G., Vance R.E., Dangl J.L. (2016) Intracellular innate immune surveillance devices in plants and animals. Science 354: aaf6395. 10.1126/science.aaf6395 27934708

[pcy185-B35] Kelley L.A., Mezulis S., Yates C.M., Wass M.N., Sternberg M.J.E. (2015) The Phyre2 web portal for protein modeling, prediction and analysis. Nat. Protoc. 10: 845. 25950237 10.1038/nprot.2015.053 PMC5298202

[pcy185-B36] Khan M., Subramaniam R., Desveaux D. (2016) Of guards, decoys, baits and traps: pathogen perception in plants by type III effector sensors. Curr. Opin. Microbiol. 29: 49–55. 26599514 10.1016/j.mib.2015.10.006

[pcy185-B37] Kourelis J., van der Hoorn R.A.L. (2018) Defended to the nines: 25 years of resistance gene cloning identifies nine mechanisms for R protein function. Plant Cell 30: 285–299. 29382771 10.1105/tpc.17.00579 PMC5868693

[pcy185-B38] Kroj T., Chanclud E., Michel-Romiti C., Grand X., Morel J.B. (2016) Integration of decoy domains derived from protein targets of pathogen effectors into plant immune receptors is widespread. New Phytol. 210: 618–626. 26848538 10.1111/nph.13869 PMC5067614

[pcy185-B39] Lahm A., Paradisi A., Green D.R., Melino G. (2003) Death fold domain interaction in apoptosis. Cell Death Differ. 10: 10. 12655289 10.1038/sj.cdd.4401203

[pcy185-B40] Launay G., Simonson T. (2008) Homology modelling of protein–protein complexes: a simple method and its possibilities and limitations. BMC Bioinformatics 9: 427. 18844985 10.1186/1471-2105-9-427 PMC2586029

[pcy185-B41] Leibman-Markus M., Pizarro L., Schuster S., Lin Z.J.D., Gershony O., Bar M., et al (2018) The intracellular nucleotide-binding leucine-rich repeat receptor (SlNRC4a) enhances immune signalling elicited by extracellular perception. Plant Cell Environ. 41: 2313–2327. 29790585 10.1111/pce.13347 PMC7266068

[pcy185-B42] Lupas A., Van Dyke M., Stock J. (1991) Predicting coiled coils from protein sequences. Science 252: 1162–1164. 2031185 10.1126/science.252.5009.1162

[pcy185-B43] Maekawa T., Cheng W., Spiridon L.N., T�ller A., Lukasik E., Saijo Y., et al (2011) Coiled-coil domain-dependent homodimerization of intracellular barley immune receptors defines a minimal functional module for triggering cell death. Cell Host Microbe 9: 187–199. 21402358 10.1016/j.chom.2011.02.008

[pcy185-B44] Maqbool A., Hughes R.K., Dagdas Y.F., Tregidgo N., Zess E., Belhaj K., et al (2016) Structural basis of host autophagy-related protein 8 (ATG8) binding by the Irish potato famine pathogen effector protein PexRD54. J. Biol. Chem. 291: 20270–20282. 27458016 10.1074/jbc.M116.744995 PMC5025708

[pcy185-B45] Maqbool A., Saitoh H., Franceschetti M., Stevenson C.E.M., Uemura A., Kanzaki H., et al (2015) Structural basis of pathogen recognition by an integrated HMA domain in a plant NLR immune receptor. Elife 4: e08709. 10.7554/eLife.08709 PMC4547098 26304198

[pcy185-B46] Meunier E., Broz P. (2017) Evolutionary convergence and divergence in NLR function and structure. Trends Immunol. 38: 744–757. 28579324 10.1016/j.it.2017.04.005

[pcy185-B47] Meyers B.C., Dickerman A.W., Michelmore R.W., Sivaramakrishnan S., Sobral B.W., Young N.D. (1999) Plant disease resistance genes encode members of an ancient and diverse protein family within the nucleotide-binding superfamily. Plant J. 20: 317–332. 10571892 10.1046/j.1365-313x.1999.t01-1-00606.x

[pcy185-B48] Meyers B.C., Kozik A., Griego A., Kuang H.H., Michelmore R.W. (2003) Genome-wide analysis of NBS-LRR-encoding genes in Arabidopsis. Plant Cell 15: 809–834. 12671079 10.1105/tpc.009308 PMC152331

[pcy185-B49] Meyers B.C., Morgante M., Michelmore R.W. (2002) TIR-X and TIR-NBS proteins: two new families related to disease resistance TIR-NBS-LRR proteins encoded in Arabidopsis and other plant genomes. Plant J. 32: 77–92. 12366802 10.1046/j.1365-313x.2002.01404.x

[pcy185-B50] Moffett P., Farnham G., Peart J., Baulcombe D.C. (2002) Interaction between domains of a plant NBS-LRR protein in disease resistance-related cell death. EMBO J. 21: 4511–4519. 12198153 10.1093/emboj/cdf453 PMC126192

[pcy185-B51] Mor�ra S., LeBras G., Lascu I., Lacornbe M.L., V�ron M., Janin J. (1994) Refined X-ray structure of *Dictyostelium discoideum* nucleoside diphosphate kinase at 1.8 A resolution. J. Mol. Biol. 243: 873–890. 7966307 10.1006/jmbi.1994.1689

[pcy185-B52] Mucyn T.S., Clemente A., Andriotis V.M.E., Balmuth A.L., Oldroyd G.E.D., Staskawicz B.J., et al (2006) The tomato NBARC-LRR protein Prf interacts with Pto kinase in vivo to regulate specific plant immunity. Plant Cell 18: 2792–2806. 17028203 10.1105/tpc.106.044016 PMC1626632

[pcy185-B53] Mundt C.C. (2018) Pyramiding for resistance durability: theory and practice. Phytopathology 108: 792–802. 29648947 10.1094/PHYTO-12-17-0426-RVW

[pcy185-B54] Narusaka M., Kubo Y., Shiraishi T., Iwabuchi M., Narusaka Y. (2009) A dual resistance gene system prevents infection by three distinct pathogens. Plant Signal. Behav. 4: 954–955. 19826224 10.4161/psb.4.10.9640 PMC2801359

[pcy185-B55] Pan Q., Liu Y.-S., Budai-Hadrian O., Sela M., Carmel-Goren L., Zamir D., et al (2000) Comparative genetics of nucleotide binding site-leucine rich repeat resistance gene homologues in the genomes of two dicotyledons: tomato and Arabidopsis. Genetics 155: 309–322. 10790405 10.1093/genetics/155.1.309 PMC1461067

[pcy185-B56] Periyannan S., Moore J., Ayliffe M., Bansal U., Wang X., Huang L., et al (2013) The gene Sr33, an ortholog of barley Mla genes, encodes resistance to wheat stem rust race Ug99. Science 341: 786–788. 23811228 10.1126/science.1239028

[pcy185-B57] Qi D., DeYoung B.J., Innes R.W. (2012) Structure–function analysis of the coiled-coil and leucine-rich repeat domains of the RPS5 disease resistance protein. Plant Physiol. 158: 1819–1832. 22331412 10.1104/pp.112.194035 PMC3320188

[pcy185-B58] Qi S., Pang Y., Hu Q., Liu Q., Li H., Zhou Y., et al (2010) Crystal structure of the *Caenorhabditis elegans* apoptosome reveals an octameric assembly of CED-4. Cell 141: 446–457. 20434985 10.1016/j.cell.2010.03.017

[pcy185-B59] Rairdan G.J., Collier S.M., Sacco M.A., Baldwin T.T., Boettrich T., Moffett P. (2008) The coiled-coil and nucleotide binding domains of the potato Rx disease resistance protein function in pathogen recognition and signaling. Plant Cell 20: 739–751. 18344282 10.1105/tpc.107.056036 PMC2329922

[pcy185-B60] Reubold T.F., Wohlgemuth S., Eschenburg S. (2011) Crystal structure of full-length Apaf-1: how the death signal is relayed in the mitochondrial pathway of apoptosis. Structure 19: 1074–1083. 21827944 10.1016/j.str.2011.05.013

[pcy185-B61] Sacco M.A., Mansoor S., Moffett P. (2007) A RanGAP protein physically interacts with the NB-LRR protein Rx, and is required for Rx-mediated viral resistance. Plant J. 52: 82–93. 17655649 10.1111/j.1365-313X.2007.03213.x

[pcy185-B62] Sandstrom A., Mitchell P.S., Goers L., Mu E.W., Lesser C.F., Vance R.E. (2018) Functional degradation: a mechanism of NLRP1 inflammasome activation by diverse pathogen enzymes. bioRxiv doi.org/10.1101/317834 10.1126/science.aau1330 PMC6532986 30872533

[pcy185-B63] Sarris P.F., Cevik V., Dagdas G., Jones J.D.G., Krasileva K.V. (2016) Comparative analysis of plant immune receptor architectures uncovers host proteins likely targeted by pathogens. BMC Biol. 14: 8. 26891798 10.1186/s12915-016-0228-7 PMC4759884

[pcy185-B64] Saur I.M.L., Conlan B.F., Rathjen J.P. (2015) The N-terminal domain of the tomato immune protein Prf contains multiple homotypic and Pto kinase interaction sites. J. Biol. Chem. 290: 11258–11267. 25792750 10.1074/jbc.M114.616532 PMC4416833

[pcy185-B65] Schreiber K.J., Bentham A., Williams S.J., Kobe B., Staskawicz B.J. (2016) Multiple domain associations within the Arabidopsis immune receptor RPP1 regulate the activation of programmed cell death. PLoS Pathog. 12: e1005769. 27427964 10.1371/journal.ppat.1005769 PMC4948778

[pcy185-B66] Su L., Quade B., Wang H., Sun L., Wang X., Rizo J. (2014) A plug release mechanism for membrane permeation by MLKL. Structure 22: 1489–1500. 25220470 10.1016/j.str.2014.07.014 PMC4192069

[pcy185-B67] Takken F.L.W., Albrecht M., Tameling W.I.L. (2006) Resistance proteins: molecular switches of plant defence. Curr. Opin. Plant Biol. 9: 383–390. 16713729 10.1016/j.pbi.2006.05.009

[pcy185-B68] Takken F.L.W., Goverse A. (2012) How to build a pathogen detector: structural basis of NB-LRR function. Curr. Opin. Plant Biol. 15: 375–384. 22658703 10.1016/j.pbi.2012.05.001

[pcy185-B69] Tameling W.I., Vossen J.H., Albrecht M., Lengauer T., Berden J.A., Haring M.A., et al (2006) Mutations in the NB-ARC domain of I-2 that impair ATP hydrolysis cause autoactivation. Plant Physiol. 140: 1233–1245. 16489136 10.1104/pp.105.073510 PMC1459841

[pcy185-B70] Tenthorey J.L., Haloupek N., L�pez-Blanco J.R., Grob P., Adamson E., Hartenian E., et al (2017) The structural basis of flagellin detection by NAIP5: a strategy to limit pathogen immune evasion. Science 358: 888–893. 29146805 10.1126/science.aao1140 PMC5842810

[pcy185-B71] Townsend P.D., Dixon C.H., Slootweg E.J., Sukarta O.C.A., Yang A.W.H., Hughes T.R., et al (2018) The intracellular immune receptor Rx1 regulates the DNA-binding activity of a Golden2-like transcription factor. J. Biol. Chem. 293: 3218–3233. 29217772 10.1074/jbc.RA117.000485 PMC5836133

[pcy185-B72] Vajjhala P.R., Ve T., Bentham A., Stacey K.J., Kobe B. (2017) The molecular mechanisms of signaling by cooperative assembly formation in innate immunity pathways. Mol. Immunol. 86: 23–37. 28249680 10.1016/j.molimm.2017.02.012

[pcy185-B73] Ve T., Williams S.J., Kobe B. (2015) Structure and function of Toll/interleukin-1 receptor/resistance protein (TIR) domains. Apoptosis 20: 250–261. 25451009 10.1007/s10495-014-1064-2

[pcy185-B74] Wang G.F., He Y., Strauch R., Olukolu B.A., Nielsen D., Li X., et al (2015) Maize homologs of hydroxycinnamoyltransferase, a key enzyme in lignin biosynthesis, bind the nucleotide binding leucine-rich repeat Rp1 proteins to modulate the defense response. Plant Physiol. 169: 2230–2243. 26373661 10.1104/pp.15.00703 PMC4634058

[pcy185-B75] Williams S.J., Sohn K.H., Wan L., Bernoux M., Sarris P.F., Segonzac C., et al (2014) Structural basis for assembly and function of a heterodimeric plant immune receptor. Science 344: 299–303. 24744375 10.1126/science.1247357

[pcy185-B76] Williams S.J., Sornaraj P., de Courcy-Ireland E., Menz R.I., Kobe B., Ellis J.G., et al (2011) An autoactive mutant of the M flax rust resistance protein has a preference for binding ATP, whereas wild-type M protein binds ADP. Mol. Plant Microbe Interact. 24: 897–906. 21539434 10.1094/MPMI-03-11-0052

[pcy185-B77] Wu C.-H., Abd-El-Haliem A., Bozkurt T.O., Belhaj K., Terauchi R., Vossen J.H., et al (2017) NLR network mediates immunity to diverse plant pathogens. Proc. Natl. Acad. Sci. USA 114: 8113–8118. 28698366 10.1073/pnas.1702041114 PMC5544293

[pcy185-B78] Wu C.-H., Krasileva K.V., Banfield M.J., Terauchi R., Kamoun S. (2015) The ‘sensor domains’ of plant NLR proteins: more than decoys? Front. Plant Sci. 6: 134. 25798142 10.3389/fpls.2015.00134 PMC4350390

[pcy185-B79] Yang J., Zhang Y. (2015) Protein structure and function prediction using I-TASSER. Curr. Protoc. Bioinformatics 52: 5.8.1–5.8.15. 26678386 10.1002/0471250953.bi0508s52 PMC4871818

[pcy185-B80] Zhang L., Chen S., Ruan J., Wu J., Tong A.B., Yin Q., et al (2015) Cryo-EM structure of the activated NAIP2–NLRC4 inflammasome reveals nucleated polymerization. Science 350: 404–409. 26449474 10.1126/science.aac5789 PMC4640189

[pcy185-B81] Zhang X., Bernoux M., Bentham A.R., Newman T.E., Ve T., Casey L.W., et al (2017) Multiple functional self-association interfaces in plant TIR domains. Proc. Natl. Acad. Sci. USA 114: E2046–E2052. 28159890 10.1073/pnas.1621248114 PMC5347627

[pcy185-B82] Zhou M., Li Y., Hu Q., Bai X-C., Huang W., Yan C., et al (2015) Atomic structure of the apoptosome: mechanism of cytochrome c- and dATP-mediated activation of Apaf-1. Genes Dev. 29: 2349–2361. 26543158 10.1101/gad.272278.115 PMC4691890

[pcy185-B83] Zhu S., Jeong R.-D., Venugopal S.C., Lapchyk L., Navarre D., Kachroo A., et al (2011) SAG101 forms a ternary complex with EDS1 and PAD4 and is required for resistance signaling against turnip crinkle virus. PLoS Pathog. 7: e1002318. 22072959 10.1371/journal.ppat.1002318 PMC3207898

